# Comparing transmission potential networks based on social network surveys, close contacts and environmental overlap in rural Madagascar

**DOI:** 10.1098/rsif.2021.0690

**Published:** 2022-01-12

**Authors:** Kayla Kauffman, Courtney S. Werner, Georgia Titcomb, Michelle Pender, Jean Yves Rabezara, James P. Herrera, Julie Teresa Shapiro, Alma Solis, Voahangy Soarimalala, Pablo Tortosa, Randall Kramer, James Moody, Peter J. Mucha, Charles Nunn

**Affiliations:** ^1^ Department of Evolutionary Anthropology, Duke University, Durham, NC 27708, USA; ^2^ Department of Sociology, Duke University, Durham, NC 27708, USA; ^3^ Marine Science Institute, University of California, Santa Barbara, CA 93106, USA; ^4^ Duke Global Health Institute, Durham, NC 27156, USA; ^5^ Science de la Nature et Valorisation des Ressources Naturelles, Centre Universitaire Régional de la SAVA, Antalaha, Madagascar; ^6^ Duke Lemur Center SAVA Conservation, Durham, NC, USA; ^7^ Department of Life Sciences, Ben-Gurion University of the Negev, Be'er Sheva, Israel; ^8^ Association Vahatra, Antananarivo, Madagascar; ^9^ UMR Processus Infectieux en Milieu Insulaire Tropical (PIMIT), Université de La Réunion, Ile de La Réunion, France; ^10^ Nicholas School of the Environment, Duke University, Durham, NC 27708, USA; ^11^ Department of Mathematics, Dartmouth College, Hanover, NH 03755, USA

**Keywords:** transmission potential networks, transmission pathways, spatial networks, superspreading potential, infectious disease transmission

## Abstract

Social and spatial network analysis is an important approach for investigating infectious disease transmission, especially for pathogens transmitted directly between individuals or via environmental reservoirs. Given the diversity of ways to construct networks, however, it remains unclear how well networks constructed from different data types effectively capture transmission potential. We used empirical networks from a population in rural Madagascar to compare social network survey and spatial data-based networks of the same individuals. Close contact and environmental pathogen transmission pathways were modelled with the spatial data. We found that naming social partners during the surveys predicted higher close-contact rates and the proportion of environmental overlap on the spatial data-based networks. The spatial networks captured many strong and weak connections that were missed using social network surveys alone. Across networks, we found weak correlations among centrality measures (a proxy for superspreading potential). We conclude that social network surveys provide important scaffolding for understanding disease transmission pathways but miss contact-specific heterogeneities revealed by spatial data. Our analyses also highlight that the superspreading potential of individuals may vary across transmission modes. We provide detailed methods to construct networks for close-contact transmission pathogens when not all individuals simultaneously wear GPS trackers.

## 1. Introduction

Infectious diseases are a major threat to human health, the global economy and international security [1]. Identifying heterogeneities in the contact patterns among host individuals is important for controlling infectious disease transmission, as this heterogeneity influences superspreading and outbreak size [2–5]. The construction and analysis of networks provide a powerful and increasingly used approach to investigate disease transmission pathways [2–5]. In contrast with compartmental models, network models take into account non-random, heterogeneous contacts between individuals [2,6]. Despite the interest in applying network science to investigate disease transmission, however, few studies have considered the types of data to use in generating networks [7,8], which can include survey questions, spatio-temporal data or proximity loggers. To capture disease transmission, the sampling methods should capture contact patterns that are relevant to the transmission mode of the infectious organism.

In network epidemiology, a network is composed of nodes, representing individuals, and edges, which quantify interactions that potentially result in pathogen transmission [2]. These interactions may include observed or reported contact events, proxies for contact such as common membership in a social group, or spatio-temporal overlap [9]. The ‘importance’ of an individual for infectious disease transmission can be assessed using various measures of centrality, which capture the number of connections an individual has (degree centrality), their connections to other highly connected individuals (eigenvector centrality) and their ability to connect disparate parts of the network (betweenness centrality) [10,11]. An individual's infection risk and superspreading potential can be quantified by their centrality, with high-centrality individuals having greater risk [4,5,12,13]. Other network measures consider the overall structure of the network, such as modularity as a measure of population subdivision [14], which affects the potential for a pathogen to successfully transmit throughout the network [15–17].

Here, we take a network-based approach to investigate potential disease transmission pathways in a rural human population in Madagascar. Infectious diseases impacting public health in Madagascar include recurring plague epidemics [18,19], large-scale measles outbreaks [20–22] and diseases associated with *Leptospira*, hantaviruses and enteroviruses such as astroviruses and coronaviruses [23–25]. Some of these pathogens are transmitted by close contact between humans, while others are environmentally transmitted through indirect contact with infected domesticated animals, wildlife or their waste products. For example, the risk of *Leptospira* transmission is greater in flooded rice fields, probably because of the environmental transmission of this water-borne bacterium [26]. For this paper, we aim to compare transmission risk between networks based on different types of connections, rather than realized infection status.

More specifically, our first aim is to investigate the estimated transmission potential of pathogens with either close contact or environmental transmission modes using spatial data that captured land use by individuals. Transmission via close contact encompasses pathogens transmitted person to person via aerosols, droplets and shared body fluids. We based our close-contact network on the probability of a dyad (pair of social contacts) coming into proximity and the probable amount of time they were in proximity. Environmental transmission refers to organisms that can be acquired through contact with environmental substrates, such as soil or water, or fomites on surfaces in homes, schools or places of worship. We used networks based on shared land use to identify potential transmission pathways of environmentally transmitted organisms. These networks assume that if a pathogen is transmitted through an environmental reservoir, then people who are in contact with the same environmental substrate are likely to be in contact with the same pathogens it contains as a function of time spent in that area [27]. Furthermore, when the transmission mechanism of a pathogen is unknown, information concerning where infected individuals are overlapping in space is key to identifying the source of an outbreak and beginning to understand the pathogen's mode of transmission [28,29].

Our second aim is to investigate how networks based on spatial proximity and shared land use, which capture key elements of transmission potential, compare to networks based on name-generating surveys, a more established approach to constructing networks. While spatial data can identify potential transmission events based on the physical proximity of individuals during a given time period, survey-based networks also have the potential to capture contact patterns, and thus potential pathogen transmission, based on the questions about who an individual contacts and in which circumstances. This may be important because GPS tracker data are time-consuming to collect and often lack social context [30]. In addition, GPS-based data require a variety of assumptions and analytical approaches to analyse. For example, when two individuals wear tracker devices at different times, it is no longer possible to simply quantify their time in proximity from the GPS coordinates and timestamps; instead, other sources of data and assumptions are needed to estimate the contact rate of that dyad.

If the networks resulting from social network surveys are similar to GPS-based networks, this may provide a way to more rapidly acquire network data or provide a bridge between data collected from people wearing GPS devices at different times. Our analyses aim to reveal which social network questions or outcomes are most informative for this purpose.

We investigated several predictions related to aim 2. We expected that edge weights (interaction intensities) and centrality metrics on the naming network generated from survey questions would covary positively with those of the GPS-based close-contact and environmental transmission networks. Specifically, we predicted that people named as spending free time with the focal subject would have stronger connections on the close-contact network generated from spatial data, while people named as working partners would best predict edges on the environmental transmission network that captured overlap in agricultural areas, such as rice fields. We also predicted that reciprocal naming on the survey, where both individuals in the dyad named each other on any of the questions, would predict higher edge weights on the other networks. Finally, we predicted that centrality on the full naming network and transmission networks would covary positively, but the naming network would miss many important, yet weak, connections between individuals, given that the participants could only name up to five other people on each of the questions.

## 2. Methods

### 2.1. Data collection

The research was conducted in the village of Mandena (14°28'36″ S 47°48'50″ E) in the SAVA region of Madagascar. Mandena is located at the edge of Marojejy National Park and serves as the gateway to the only tourist-accessible region of the park. From the park, the habitat follows a degradation gradient from semi-intact forest to secondary forest, then brushy fallow fields, and agricultural plots leading to the village. The village is roughly 1 km^2^ in size and is home to approximately 2700 people (based on census data from local authorities), and there is little immigration or emigration from villages in this region [28]. The primary occupation in Mandena is agriculture, with most people engaging in subsistence crop (rice) and vanilla farming [31].

Data collection took place during the transitional period from the dry to the wet season over the course of 7 weeks from October to mid-November 2019. We conducted 176 social network surveys of adults aged 18 years or older. The survey was administered by J.Y.R. and conducted in the local Malagasy dialect. Participants included women (*n* = 67) and men (*n* = 109) ranging in age from 18 to 82, with a mean age of 41.8 ± 15.1 (table 1). We used a ‘snowball sampling’ technique in which name-generating questions from one round of surveys were used to create a list of individuals to survey in the next round of surveys [32]. Name-generating questions were based on a recent social network study [33] in which respondents were prompted to name up to five other people who: (i) they meet in their free time, (ii) they would ask to help them on their farmland, (iii) would come to them to get help on their farmland, (iv) they would ask if they urgently needed food, and (v) would come to them if in urgent need of food (table 2). Questions were pilot tested within the community and adjusted accordingly to ensure that they were culturally appropriate and that they captured deeper relationships and sustained interactions.

**Table 1.** Demographic summary of individuals named, surveyed, or surveyed and wore a GPS tracker during the study. All network comparisons included in this study are limited to the individuals who wore a GPS tracking device.

	surveyed (*n* = 176)^a^		GPS (*n* = 123)	
	female	male	female	male
% (*n*)	38.1 (67)	61.9 (109)	44.7 (55)	55.3 (68)
age (years)				
mean ± s.d.	44.9 ± 14.7	39.9 ± 15.1	45.8 ± 15.2	43.1 ± 15.5
(range)	(18, 82)	(18, 79)	(18, 82)	(18, 79)
have a partner				
% (*n*)	62.7 (42)	83.5 (91)	61.8 (34)	83.8 (57)
main activity, % (*n*)				
crop farmer	73.1 (49)	56.9 (62)	69.1 (38)	55.9 (38)
mixed crop and livestock	23.9 (16)	42.2 (46)	27.3 (15)	42.6 (29)
other	3.0 (2)	9.2 (1)	3.6 (2)	1.5 (1)
education, % (*n*)				
higher	4.5 (3)	15.6 (17)	3.6 (2)	13.2 (9)
secondary	23.9 (16)	21.1 (23)	27.3 (15)	26.5 (18)
primary	67.1 (45)	56.9 (62)	63.6 (35)	51.5 (35)
none	4.5 (3)	6.4 (7)	5.5 (3)	8.9 (6)

^a^Of all people named (*n* = 745) during the course of this study, 40.4% (*n* = 301) were female and 59.6% (*n* = 444) were male.

**Table 2.** Name-generating questions asked in the social network survey. The responses to all questions were grouped to form a ‘full naming network’, and subsets of the questions were grouped to form ‘free time’ (question 1), ‘farming’ (questions 2 and 3) and ‘food’ (questions 4 and 5) networks.

naming network		question
full	free time	1. Please list the first and last names of 5 people who you meet with in your free time.
	farming	2. Please list the first and last names of 5 people who would help you if you need help in your farmland if you want to finish it fast.
		3. Please list the first and last names of 5 people who come to you for help in their farmland if they want to finish work fast.
	food	4. Please list the first and last names of 5 people you would go to if you urgently needed rice or other groceries.
		5. Please list the first and last names of 5 people who could come to you if they urgently need rice or other groceries.

Over the same time period, we distributed GPS trackers (iGot-U 120; Mobile Action, New Taipei City, Taiwan) to consenting participants (*n* = 134) after they completed the name-generating survey. The final GPS dataset contained 123 individuals (i.e. 7503 unique pairs of individuals), with some attrition due to device malfunction or participant non-compliance. The mean age of participants for whom we obtained GPS data was 44.38 ± 15.33 (table 1). The devices were distributed to participants on Fridays and Saturdays and collected one week later. We excluded the day of distribution from the analysis to adjust for behavioural patterns that were generated through interaction with our research team. Participants were instructed to wear the GPS tracker at all times and to set it close by during times such as sleeping or bathing when they were not wearing the device. To make the device easier to wear, it was attached to a lanyard. We also instructed participants to inform us if they forgot to wear the device so we could remove that period from the dataset. The device recorded the participant's location every 3 min until the devices were returned or the battery died (mean duration of function was 5.2 ± 1.2 days). The average reported location estimation accuracy across varying degrees of cover for the iGot-U 120 is 9.2 m (±0.2 m) [34].

### 2.2. Data preparation

All data preparation and analyses were completed in R v. 4.0.5 [35].

#### 2.2.1. Social ‘naming’ network

We assigned a unique identifier to each person named by an interviewee during the name-generating portion of the survey. Individuals who were named and subsequently surveyed during our study were assigned a definitive identifier, while those who were named but not surveyed, owing to our limited time frame or them choosing not to participate, were assigned an identifier based on their name, known nicknames and gender with help from a community member. Care was taken to assign the same identifier to individuals named by multiple interviewees. For these analyses, we excluded individuals who were not surveyed and surveyed individuals who did not opt to wear a GPS tracker. We generated directed and undirected social networks based on who named each other in the survey using the package *igraph* [36]. We created a ‘full’ network using all the names an interviewee listed regardless of the question, a ‘free time’ network (question 1), a ‘farming’ network (questions 2 and 3) and a ‘food’ network (questions 4 and 5). Edges were weighted by the number of times individuals named each other (e.g. a weight of 1 if an individual named the person once). For undirected network representations, we summed the directed edge weights between the two individuals (range 1–10). To identify reciprocated naming edges, we used the *igraph::which_mutual* function on the full directed network to identify dyads where both individuals named the other regardless of the question.

#### 2.2.2. GPS data preparation

We focused on interactions that occurred in Mandena and the immediately surrounding area. GPS tracker data were thus cleaned to remove fixes that were determined to be inaccurate, recorded outside our study area or were recorded on days the participant did not wear the GPS. Specifically, we defined our study area by selecting a boundary that maximized the number of points included and minimized the total area. We created the boundary by calculating the minimum convex polygon (MCP) [37] for all GPS data from 90% to 100% in 0.5% to 0.01% increments using *adehabitatHR::mcp.area* [38]. This resulted in a 63.1 km^2^ sized grid with 10 m^2^ cells that included 94.64% (584 871/617 968) of the recorded locations. To account for days when participants did not wear the GPS (i.e. left the tracker at home), we calculated the daily 99% MCP and excluded days for which the entire 99% MCP fell within 1 ha, based on two assumptions: (i) everyone leaves their house regularly to access water for bathing, outdoor latrines and agricultural land outside the village (K Kauffman & CS Werner 2019, personal observation); (ii) studying the trajectories of individuals with less than 1 ha MCP, we found that they followed a ‘starburst’ pattern, indicating that the total area was likely to be the result of the scatter of inaccurate GPS points. This resulted in the removal of 26.4% (154 675/584 871) of the remaining data points. This resulted in a dataset containing 123 individuals (7503 dyads) and 1115 days of GPS data with a mean of 9.1 ± 6.3 days (2.1 ± 1.2 weeks) of GPS data per individual (range 1–28 days, 1–6 weeks).

To quantify space use across the landscape, we estimated the home range and usage probability, or utilization distributions (UDs), for each individual using a dynamic Brownian Bridge Movement Model [39] in the *move* package [40]. To quantify each pair of individuals' time-independent interactions, we calculated the volume of intersection (VI) using a dBBMM-suited adaptation of the *overlap* function in *adehabitatHR* [38,41]. We chose to use VI instead of the UD overlap index because it is better suited to non-uniformly distributed UDs with a high degree of overlap. We assumed for the overlap calculations that individuals had similar UDs week to week and used the entirety of each individual's GPS data to calculate their UD. We tested this assumption using 14 individuals for whom we had more than 10 days of GPS data spanning at least three weeks by calculating a UD for each week that the individual wore a GPS and computing the VI of their home range (95% UD) between weeks for each individual. We found that the mean VI from week to week of all these individuals (*n* = 14) was 66.68% ± 6.74%.

#### 2.2.3. Close-contact networks

For individuals who wore a GPS on the same day(s), we calculated the close-contact rate or proportion of proximal GPS fixes out of all simultaneous fixes. We used a distance threshold of 17.04 m to define a proximal contact, which captures 98% of true positive contacts given the location accuracy of the iGot-U 120, using the *findDistThresh* function of the *contact* package in R [42]. We defined simultaneous fixes as all fixes recorded within 1 min 30 s (half the sampling window) of each other. We also calculated the number of fixes where the pair remained in continual contact (e.g. consecutive fixes that were also proximal), the mean length of contact time (assuming that all contacts, including instantaneous contacts, were at least equal to the temporal threshold) and the total time elapsed of all continual contacts. The observed close-contact rate between individuals wearing a GPS at the same time only provided a snapshot of the full network because different sets of individuals wore devices each week and therefore unobserved edges could reflect unobserved contacts (e.g. either or both individuals were not wearing a GPS during a contact) or true absences. The full close-contact network was imputed from the observed close-contact network, using all dyads that had a minimum of 240 simultaneous fixes (44.9%, *n* = 3369).

We imputed the missing edges (55.1%; *n* = 4134) and all the edge weights with a two-step analysis: first, we used an exponential random graph model (ERGM) [43–45] to determine whether an edge existed; next, we multiplied the probability an edge exists by its edge weight, as predicted by the general linear model (GLM). We give details on these steps in the next three subsections.

#### 2.2.4. Exponential random graph model-based edge imputation

We used the *ergm* package [46,47] to fit the ERGM, assess model convergence and simulate edges. Because the ERGM was intended to predict edges between all participants, not just participants who wore a GPS tracker during the same week, we pooled all of an individual's GPS trajectories at the scale of a single week (pooled proximity), then recalculated proximity using the methods described above. We used pooled proximity to create a binary network, where the upper 50% of values were assigned an edge. This 50th percentile contact rate corresponded to approximately one contact per day.

We then fitted the ERGM by using the following edge covariates: VI of the home range (95%) and core-use area (50%), separate VIs of the home range at night and during the day; the interaction of distance between individuals' houses and whether they lived less than 25 m apart; the weighted full, undirected naming network (weights range 1–10); whether naming was reciprocal. We also included nodal covariates for gender match and age difference, as well as the structural terms edges, geometrically weighted edgewise-shared partner (GWESP), and geometrically weighted non-edgewise-shared partner (GWNSP) [46]. We used a Markov chain Monte Carlo (MCMC) interval of 1000, a burn-in of 70 000 and a maximum of 10 000 iterations to fit each model, then determined model fit using *ergm::mcmc.diagnostics* [48] and examining diagnostic convergence plots. We ranked all models based on the Akaike information criterion (AIC) values and predictive accuracy using the held-out predictive evaluation method for cross-validation [49]. To implement this, we randomly sampled 80% of observed edges from the observed proximity network as a training dataset and simulated 500 networks using the held-out 20% of observed edges. Then, we calculated the sensitivity, specificity and predictive accuracy of each model (electronic supplementary material, table S1).

After identifying the best-performing ERGM, we simulated 1000 complete interaction matrices using the *ergm::simulate* function with observed proximity as the basis network.

#### 2.2.5. General linear mixed model edge weights

We predicted the edge weights (e.g. proportion of close contacts) using GLM models. Fixed effects were the same nodal and edge covariates used in the ERGM. We modelled the observed close-contact rate between two individuals as a function of their VI calculated from GPS fixes recorded when both individuals were wearing a tracker and compared this with models using separate VIs of the home range at night and during the day. The data for the response (proportion of proximal contacts) and predictors (VI, naming, house distance and age difference) were right-skewed and zero-inflated; to address this, we modelled the data using a Tweedie distribution with the index parameter between 1 and 2 [50].

We ranked all model combinations by AIC using the *MuMIN* package [51]. To assess the sensitivity, specificity and predictive accuracy of the top models, we trained the models using 80% of the data from pairs of individuals who wore the GPS at the same time and tested on the withheld 20% of the data. The threshold for the accuracy measures was determined using the *ROCR* package [52]. Using the top model by predictive accuracy, we estimated proportions of close contacts between all pairs (*n* = 7503), including individuals who did not wear a GPS at the same time, by using the VI of each dyad's complete UD, instead of the subset VI used to build the model.

#### 2.2.6. Simulated close-contact network

We then multiplied the 1000 simulated interaction matrices derived from the ERGM by the interaction matrix derived from the GLM to weight the probability of edge presence by the expected contact rates. This resulted in a distribution of 1000 full close-contact networks.

#### 2.2.7. Environmental overlap network

To determine the co-use of different land-use areas, and thus sites of potential transmission of environmental pathogens, we classified the habitat around the village from satellite imagery. We searched for low cloud cover (0–5%) Sentinel-2 satellite imagery over the area of interest from October 2019 to October 2020. We used a 10-m-resolution image dated 14 July 2020 and built a composite image using the first nine bands. Areas within and immediately surrounding the village, large rice fields and water sources (priority areas) were manually divided into polygons based on land-cover type. Rice fields and water sources are permanent, although there may be slight seasonal variation in their size. Additional training samples were then created for each of seven land-use categories (primary forest, secondary forest, rice, brushy regrowth, village, water and bare ground) and were used to perform supervised classification of the rest of the landscape using a support vector machine model in ArcGIS Pro (v. 2.5.0). An accuracy assessment performed on the pre-converted raster showed 84% accuracy (*κ* = 0.785, *n* = 500 points generated from stratified random sampling). We also verified accuracy by investigating the land-type composition using polygons created by visiting discrete land-cover areas (i.e. a rice field or secondary forest patch) and creating GPS traces of their perimeter. Results showed that, on average, 89% of the area covered by rice polygons was classified as rice, and 93% of the area in secondary forest polygons was classified as such (electronic supplementary material, table S2).

We created discrete land-use areas by overlaying a grid with cells sized 30 × 30 m over the study area. For each grid cell, we counted the number of pixels that were classified as each land-cover class and calculated the proportion of each individual's home range (95% UD) that was spent within each grid cell using the *exact_extract* function in the *exactextractr* package [53]. To control for the random effect of the grid position, we repeated this process nine times, shifting the grid location by 10 m each time to cover all unique grid locations, then took the average values for each grid cell. We then created a bipartite network of individuals and grid cells, using time spent in a location (proportion of UD) as edge weights; therefore, interactions were scaled to the time a person was exposed to that substrate. This network was then divided into subsets by land-use category (rice, water and village) to aid in describing shared habitats relevant to the transmission modes of different pathogens. Finally, we created a unipartite projection of individuals connected by shared polygon spaces weighted by the sum of the product of the dyad's UD proportions in each grid cell.

### 2.3. Statistical analysis

For all network-wide comparisons, we used *igraph* functions: *is.connected, diameter, average.path.length, reciprocity, graph.density, transitivity* and *modularity* [36,54–57]. We compared modularity between all the networks to assess the overall modularity of each network, with communities detected via the Louvain method [58]. To compare individuals' positions on each network, we calculated their eigenvector (Pagerank for the directed naming network), strength and betweenness centrality on each network [10,55,59–61]. To calculate these statistics for the close-contact network, we calculated the aforementioned metrics on each of the 1000 simulated networks and used the median values for further analysis. We then investigated correlations among centrality metrics on these networks using Spearman's rank correlation test. We used the Wilcoxon rank-sum test to investigate differences in edge weights between dyads who did not name each other, named each other and reciprocally named each other. For the close-contact network edge weight comparison, we used the mean predicted edge weights. For all tests, the alpha level was 0.05.

## 3. Results

The demographic profiles of those who did and did not wear GPS devices were similar (table 1). Of those named in the survey, 40.4% (*n* = 301) were female, which was comparable to our surveyed (38.1%, *n* = 67) and GPS-wearing subpopulations (44.7%, *n* = 55). The mean age of survey participants was 41.8 ± 15.1 years and that of participants who chose to wear a GPS was 44.3 ± 15.4 years. The ages of participants in both groups ranged from 18 to 82 years. Farming was the reported main activity of 98.3% of survey participants (*n* = 173) and 97.6% of participants who chose to wear a GPS (*n* = 120). Education attainment of survey participants was similar to that of participants who chose to wear a GPS with, respectively, 60.8% (*n* = 107) and 56.9% (*n* = 70) having up to a primary education and 33.5% (*n* = 59) and 35.8% (*n* = 44) having a secondary or higher education.

### 3.1. Imputing the close-contact network

Edges in the close-contact network were best predicted by the ERGM that includes the covariates VI of the dyad's home range at night and during the day, the interaction of the distance between their houses and if they lived less than 25 m apart, gender match and age difference, along with the structural terms edges, GWESP and GWNSP. The chosen model had a sensitivity of 0.54, specificity of 0.89 and accuracy of 0.78 (electronic supplementary material, table S1). Among the 1000 simulated networks, 6.49% (*n* = 474) of edges were present in every simulation and 1.63% (*n* = 125) of edges were not present in any simulations. A model containing edges, the undirected full naming network, if their naming was reciprocated, house distance, GWESP and GWNSP had a 16.2 point lower AIC. However, the edge predictions were highly correlated with those of the model not containing the naming data (Spearman's *ρ* = 0.97222, *p* < 0.001; electronic supplementary material, table S1), so we used the model without naming data in further analyses.

Proximity was best explained by the GLM that included the cube root of the VI of the home ranges (95%), the cube root of the VI of the core-use area (50%), the distance between individuals' houses and the gender match term. Models (*n* = 3) containing the above predictors and either the difference in age between individuals, if individuals lived within 25 m of each other, or the number of times individuals named each other also had substantial support (ΔAIC < 2). The chosen model was the simplest, and all predictors were present in all the other models with substantial support. This model has a sensitivity of 0.88, specificity of 0.75 and accuracy of 0.80 using a threshold of 0.0016, which is equivalent to slightly less than one contact per day (0.768).

### 3.2. Network-wide comparisons

**Figure 1.**
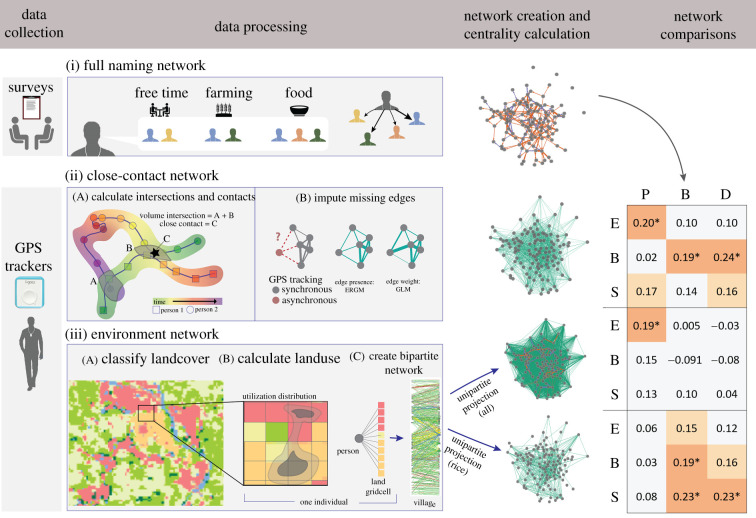
Workflow schematic and network comparisons. (i) Name-generating surveys were used to form a ‘full naming network’ based on survey questions regarding free time, help with farming and help with food. (ii) Survey participants also wore a GPS tracker from which we inferred a close-contact network. Given that participants work GPS trackers at different times, we inferred close contacts (ii.A) using a pseudo-hurdle model (ii.B) where the probability of edge presence was determined using an ERGM and edge weight was determined using a GLM. We also calculated an environmental overlap network (iii) by first classifying land cover based on GPS imagery (iii.A). We calculated the proportion of time that each person spent in a given grid cell of a given land-cover class (iii.B). We then used these data to create a bipartite network of all shared spaces (iii.C), as well as a sub-network of flooded rice field co-use to demonstrate land-cover specific overlap. Finally, we calculated the unipartite projection for each of these environmental overlap networks. Eigenvector (E), betweenness (B) and strength (S) centrality were calculated on all GPS-based networks. Pagerank (P) centrality replaced eigenvector centrality on the naming network to account for edge directionality. We used Spearman rank correlations (*ρ*) between the full naming network and each GPS tracker-based network to compare each participant's relative importance on each network (§3.3). Significant correlations (*p* < 0.05) are indicated by an asterisk. Final network graphs are provided in high resolution in electronic supplementary material, figure S1.

The resulting full naming network for the 123 individuals who wore a GPS is shown in figure 1 and electronic supplementary material, figure S1a. This network is disconnected because four individuals were named by survey participants who did not wear a GPS, and they subsequently named individuals who either did not wear a GPS or were not surveyed. The close-contact network was strongly connected in 13.2% (132/1000) of simulations; the mean network is shown in figure 1 and electronic supplementary material, figure S1b.

The entire environmental overlap network (figure 1 and electronic supplementary material, figure S1c) was strongly connected, and the flooded rice fields environmental overlap network (figure 1 and electronic supplementary material, figure S1d) had four disconnected nodes. We would expect environmentally transmitted pathogens to potentially spread most quickly on the environmental overlap networks because of the relatively high transitivity and low modularity of these networks (table 3) [62]. However, when the bipartite nature of these networks is taken into account, the modularity of the flooded rice field overlap network increases substantially from 0.55 to 0.75, demonstrating the importance of incorporating locations into networks of shared land use (electronic supplementary material, table S3).

**Table 3.** Network characteristics comparisons.

network	diameter	average distance	density	transitivity	modularity (Louvain)
full naming network, directed	22	5.2	0.02	0.16	0.71^a^
close contact^b^	0.12 ± 0.05	1.77 ± 1.02	0.27 ± 0.01	0.47 ± 0.01	0.63 ± 0.00
environmental, full	<0.01	1.03	0.97	0.98	0.54
environmental, rice	<0.01	1.41	0.59	0.76	0.55

^a^Calculated on an undirected network.

^b^The mean ± the standard deviation for each of the 1000 simulated close-contact networks.

The modularity of the close-contact network (0.63 ± 0.003) is higher than the unipartite projection of the flooded rice field network but lower than its bipartite projection (table 3). The naming network has the lowest density (0.02) and transitivity (0.16) and the highest modularity (0.71); thus, we would expect pathogens to transmit more slowly using this network alone. Descriptions of the bipartite environmental overlap and all naming networks based on subsets of the questions (whom you spend your free time with, whom you help and who helps you in their/your field, whom you help and who helps you if they/you need food) can be found in the electronic supplementary material, table S3.

### 3.3. Correlations in centrality

Correlations in eigenvector centrality (Pagerank for directed naming network) ranged from −0.21 to 0.72. Strength centrality (degree for directed naming network) correlations ranged from −0.21 to 0.73 and betweenness centrality ranged from −0.09 to 0.19. Spearman rank correlations were low when comparing the measures of centrality on the close-contact and environmental networks with centrality on the naming network (figure 1). The mean of the absolute values of the correlation coefficient (*ρ*) was 0.13 ± 0.07 (range 0.005–0.24). Pagerank centrality on the naming network was significantly correlated with eigenvector centrality on the entire environmental network (0.19, *p* = 0.03) and close-contact network (0.20, *p* = 0.03). Betweenness centrality on the naming network was significantly correlated with betweenness centrality on the close-contact network (0.19, *p* = 0.04) and betweenness centrality (0.19, *p* = 0.027) and strength centrality (0.23, 0.01) on the rice fields environmental network. Degree on the naming network was correlated with betweenness on the close-contact network (0.24, *p* = 0.01) and strength on the rice network (0.23, *p* = 0.01).

We consider individuals with the highest centrality (top 10%) for each centrality metric by network as the potential superspreaders on the given network. Across all metrics and networks, 58% (71/123) of individuals were identified as a potential superspreader at least once. Of those 71 individuals, 23 individuals are particularly strong suspects for being potential superspreaders because they were high-centrality individuals on two (*n* = 18, 25%) or three (*n* = 5, 7%) networks regardless of which centrality metric was used.

### 3.4. Reciprocal naming and edge weights

**Figure 2.**
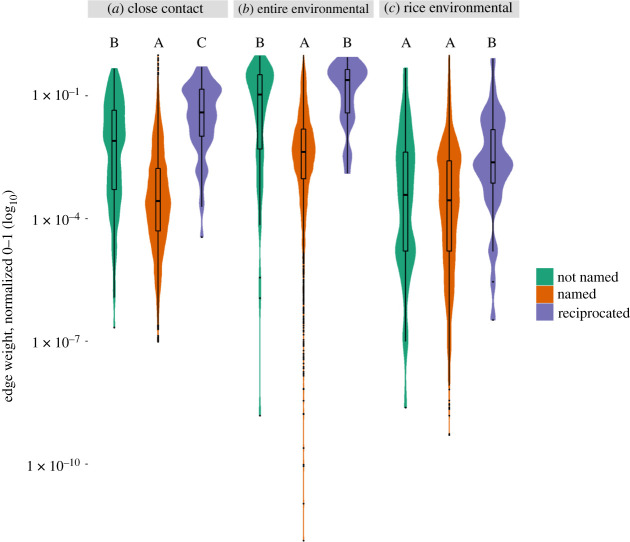
Edge weights on GPS-derived networks were higher between individuals who named each other in social network surveys. Edge weights connecting individuals on the (*a*) close contact, (*b*) entire environmental and (*c*) flooded rice field environmental networks were significantly higher when both individuals named one another (reciprocated) for any of the survey questions. Post hoc comparisons were conducted using the Wilcoxon rank-sum test with an alpha level of 0.05.

Each of our networks with 123 nodes inherently includes 7503 dyads (e.g. possible edges). The full naming network consists of 176 edges where only one individual in a dyad named the other (named edge) and 66 edges where both individuals in the dyad named each other (reciprocated edge), resulting in a reciprocity of 0.43. Edge weights in the close-contact network were significantly higher if that edge was named (*p* < 0.001) or reciprocated (*p* < 0.001) than if it was not named in the naming network (figure 2*a*). The close-contact edge weights of reciprocated edges were also significantly higher than the named edges (*p* < 0.001; figure 2*a*). Edge weights on the entire environmental overlap network were also significantly higher if named (*p* < 0.001) or reciprocated (*p* < 0.001) than if not named, but reciprocated edges did not have a significantly higher weight than non-reciprocated edges (*p* = 0.095; figure 2*b*). For the flooded rice field environmental overlap network, edge weights were significantly higher if the edge was reciprocated than if it was not named (*p* < 0.001) or named (*p* = 0.015), but no significant differences were found between not named and named edges (*p* = 0.51; figure 2*c*).

We repeated the above comparisons for the specific questions in table 2, predicting that the free time question (1) would covary most strongly with the close-contact network and the farming questions (2 and 3) would covary most strongly with the flooded rice field environmental overlap network. We found that on the close-contact network the greatest difference in mean edges weights (range 9.75 × 10^−8^ to 1.0) was between the not named and reciprocally named groups on the food network (questions 4 and 5), with the mean edges weight of the reciprocated edges being 0.161 higher than the not named edges (*p* < 0.001). This was followed by a difference of 0.114 between the reciprocally named and not named edges on the free time (question 1) network (*p* < 0.001). On the entire environmental overlap network, the greatest difference in mean edge weights (range 1.88 × 10^–13^ to 0.138) was again between the reciprocal named and not named edges on the food network (0.061 higher, *p* < 0.001), followed by the free time network (0.058, *p* < 0.001). Most of the differences in edge weights (range 3.91 × 10^−14^ to 7.61 × 10^−5^) among the three groups were not significant on the flooded rice field overlap network; however, the greatest significant difference was between reciprocal named edges and not named edges on the farm network (6.05 × 10^−6^, *p* = 0.0015).

The outliers with high edge weights in the close-contact and environmental overlap networks that were present (e.g. not named) on the full naming network were investigated further. We found across these networks that the outliers had significantly higher VI of their core-use areas (*p* < 0.0001) than the non-outliers. On the close-contact network, the outliers had a mean VI of their 50% UD of 2.56 ± 6.30 compared with 0.0004 ± 0.014 for the non-outliers. On the entire and flooded rice environmental overlap networks, the mean VI was, respectively, 2.90% and 1.17% higher. This aligned with outliers living on average closer together than non-outliers (*p* < 0.001) on the three networks. The mean distance between houses of outliers was 113 m less than non-outliers on the close-contact network, on the entire environmental overlap network was 142 m less and on the rice environmental overlap network was 35 m less. Outliers were also more likely to live less than 25 m apart from each other than non-outliers (*p* < 0.05). No significant differences were found between dyads of the same gender versus dyads of different genders (*p* > 0.05) on the three networks.

## 4. Discussion

Integrating spatial and social network-based information into the analysis of disease transmission pathways enables better prediction of when and where transmission events occur [63–65]. Our study shows that the networks based on surveys are not perfectly comparable to GPS-derived networks based on close contacts and shared space use. We found only weak, positive correlations between centrality metrics on the different networks, and individuals exhibiting the highest centralities often differ across networks, suggesting that predictions for superspreading potential would also vary [4,5,12,13]. Yet we also discovered that naming and reciprocal naming within a dyad predicted significantly higher edge weights in the corresponding close-contact and entire environmental overlap networks (figure 2), demonstrating that some signatures of strong connections based on social surveys also predict transmission-relevant overlap. The connections identified in the social network were important on the close-contact and environmental transmission potential networks. However, the structure of these networks was not fully captured using the social network surveys owing to many missed strong and weak connections. The structural differences between these networks highlight the importance of GPS tracker data to capture direct interactions between people and indirect interactions via the co-use of spaces that are relevant to pathogen transmission [63,66].

Identifying dyads who showed discordant connections in survey and GPS data provides insight into the contact heterogeneities that are captured by GPS-based networks. We found that individuals with a high degree of overlap who did not name each other were more closely associated with each other spatially, as measured by a high VI of their core-use areas (50% UD) and living closer together. We expect transmission potential within a household to be high and thus within-household edge weights in all our networks to be higher. However, given the density of homes in the village and the accuracy of the GPS tracking devices we used, it is likely that we also imputed that neighbours had a higher edge weight on the close-contact network and the entire environmental overlap network. Reflecting on these findings given the name-generating questions we asked (table 2), we suspect that participants were not naming individuals in their household because the questions asked about circumstances in which cohabitants would likely be in the same circumstance as the participant and therefore not someone they would go to or would come to them for help. Furthermore, participants might not have listed household members as people with whom they spend their free time, instead of naming friends outside of the home.

Previous studies comparing networks as described by participants based on their social connections or recall of close contacts are limited in their ability to describe transmission because numerous weak connections between individuals are missed [8]. Furthermore, participants' descriptions of interaction strength are influenced by their perceptions, as shown by the low reciprocity on our full naming network (0.43), which is a common phenomenon on social networks [67]. Spatio-temporal data-based network studies are limited in that participants need to simultaneously wear trackers, and the fix rate, or the resolution of the tracker, needs to be extremely precise to capture true contacts (see [42]). We were able to expand the time frame in which GPS tracker data can be used by implementing spatial ecology methods to estimate where individuals are likely to be located. The resulting close-contact network was much denser and less modular than the full naming network. Likewise, the entire environmental overlap network and flooded rice field overlap networks were also denser and less modular. The potential ‘superspreaders’ on these networks (e.g. high-centrality individuals) were mostly different across networks, with 18.7% (23/123) of individuals being identified as a superspreader regardless of the centrality metric used on more than one network.

To model a specific pathogen of interest additional factors, such as the effects of the seasonality of pathogen reservoirs and satellite imagery, resolution and accuracy of GPS tracker data, and temporal thresholds for contacts should be considered. The major limitations to our study arise from participants wearing GPS trackers during different weeks of the study. To overcome this, we assumed no seasonal variation in our study period and that individuals exhibited regular movements, or high fidelity, from week to week, which we tested and found support for by comparing the VI of individuals' home ranges between weeks [41]. However, in doing so, we introduced more uncertainty into our GPS-based networks. Likewise, our close-contact network does not provide a description of the actual number of times a pair of individuals was in close proximity; instead, the network provides a probability that a pair came into contact and the predicted contact rate. The distance threshold we used to determine when a dyad was in proximity probably overestimated the contact rates between individuals. Conversely, the temporal resolution at which GPS fixes were recorded and removal of erroneous points probably resulted in missing brief contacts and underestimating the duration of contacts.

Additional limitations are also worth noting. Non-compliance with the use of the GPS created a challenge because it resulted in excluding days from our analysis. Our exclusion method based on the area traversed in a calendar day potentially excluded days from our analysis when the participant was wearing the GPS device and truly not moving from their house. However, based on observations of daily routines in rural Madagascar, we are comfortable assuming everyone leaves their house daily and were able to validate this by identifying a ‘starburst’ pattern in GPS tracks from those days, indicating the total area was likely to be due to the scatter of inaccurate GPS points. In addition, a mismatch may occur between the naming and GPS-based networks because we collected GPS data during a single season and participants probably did not limit the people they named during the survey to the same time frame. Lastly, we only had data on about 10% of adults living in the village and no children, who have been identified in other studies as playing an important role in close-contact transmission [68].

## 5. Conclusion

Simultaneous spatial and social network data provide a more complete and more complex framework to study disease transmission potential and build a framework to investigate the spatial and social heterogeneities of pathogen transmission [63]. We have shown that networks representing GPS tracker-based close contact and environmental overlap in select land-use areas identify different central individuals and important connections between individuals, thus capturing heterogeneities in contact patterns that are relevant to pathogen transmission. The many strong and weak connections that are missed when using survey data alone are likely to be important to pathogen community structure and transmission. Thus, social network surveys provide context to understanding disease transmission pathways but do not substitute for spatial data. Future directions include incorporating data on the infection status of individuals with directly and environmentally transmitted pathogens to validate these networks.

## Data accessibility

All code is available at https://github.com/MadagascarEEID/Compare-TPN-Mandena1. Data are available in the electronic supplementary file; however, location data are unavailable as they are personally identifiable.

The data are provided in the electronic supplementary material [69].
